# Light-evoked Somatosensory Perception of Transgenic Rats That Express Channelrhodopsin-2 in Dorsal Root Ganglion Cells

**DOI:** 10.1371/journal.pone.0032699

**Published:** 2012-03-06

**Authors:** Zhi-Gang Ji, Shin Ito, Tatsuya Honjoh, Hiroyuki Ohta, Toru Ishizuka, Yugo Fukazawa, Hiromu Yawo

**Affiliations:** 1 Department of Developmental Biology and Neuroscience, Tohoku University Graduate School of Life Sciences and JST, CREST, Sendai, Japan; 2 Tohoku University Basic and Translational Research Centre for Global Brain Science, Sendai, Japan; 3 Department of Physiology, National Defense Medical College, Tokorozawa, Japan; 4 Department of Anatomy and Molecular Cell Biology, Nagoya University Graduate School of Medicine, Nagoya, Japan; 5 Center for Neuroscience, Tohoku University Graduate School of Medicine, Sendai, Japan; University of Cincinnatti, United States of America

## Abstract

In vertebrate somatosensory systems, each mode of touch-pressure, temperature or pain is sensed by sensory endings of different dorsal root ganglion (DRG) neurons, which conducted to the specific cortical loci as nerve impulses. Therefore, direct electrical stimulation of the peripheral nerve endings causes an erroneous sensation to be conducted by the nerve. We have recently generated several transgenic lines of rat in which channelrhodopsin-2 (ChR2) transgene is driven by the Thy-1.2 promoter. In one of them, W-TChR2V4, some neurons were endowed with photosensitivity by the introduction of the ChR2 gene, coding an algal photoreceptor molecule. The DRG neurons expressing ChR2 were immunohistochemically identified using specific antibodies to the markers of mechanoreceptive or nociceptive neurons. Their peripheral nerve endings in the plantar skin as well as the central endings in the spinal cord were also examined. We identified that ChR2 is expressed in a certain population of large neurons in the DRG of W-TChR2V4. On the basis of their morphology and molecular markers, these neurons were classified as mechanoreceptive but not nociceptive. ChR2 was also distributed in their peripheral sensory nerve endings, some of which were closely associated with CK20-positive cells to form Merkel cell-neurite complexes or with S-100-positive cells to form structures like Meissner's corpuscles. These nerve endings are thus suggested to be involved in the sensing of touch. Each W-TChR2V4 rat showed a sensory-evoked behavior in response to blue LED flashes on the plantar skin. It is thus suggested that each rat acquired an unusual sensory modality of sensing blue light through the skin as touch-pressure. This light-evoked somatosensory perception should facilitate study of how the complex tactile sense emerges in the brain.

## Introduction

Knowledge of the world is obtained exclusively via perception through our sensory systems which consist of peripheral sensory organs, sensory nerves and the central nervous system (CNS). In principle, a sensation is classified according to its modality, that is, the kind of energy inducing physiological transduction in a specific group of sensory organs. For example, in the somatosensory systems, each mode of touch-pressure, temperature or pain is sensed by sensory endings of different dorsal root ganglion (DRG) neurons. Their signals are conducted to a specific cortical locus as nerve impulses, which are then integrated to generate somatosensory perception. Therefore, non-physiological energy transduction such as direct electrical stimulation of a peripheral nerve causes an erroneous sensation to be conducted by the nerve.

In the case of light, rhodopsins are molecules involved in its perception by the photoreceptor cells in the vertebrate retina [1], [2]. Each rhodopsin is a seven-pass transmembrane molecule, homologous to G-protein-coupled receptors, and activates cyclic GMP phosphodiesterase upon activation. With the subsequent reduction of the intracellular level of cGMP, the cyclic-nucleotide-gated cation channels are closed [3], [4]. A light signal is thus converted into an electrical one through a cascade of at least four molecules. On the other hand, light is perceived by archaea-type rhodopsins, channelrhodopsin-1 (ChR1) and -2 (ChR2), during the light-directed behavior of a unicellular green alga, *Chlamydomonas reinhardtii* [5]–[9]. Each channelrhodopsin consists of a seven-pass transmembrane apoprotein and a retinal which covalently binds to it. The photoisomerization of all-*trans*-retinal to the 13-*cis* configuration is coupled to conformational changes in the protein that result in increased cation permeability. A light signal is thus converted into an electrical one by a single molecule [10]. Previously, it was reported that neurons were endowed with sensitivity to blue light by introduction of the ChR2 gene [11]–[13]. This optogenetic method has become a powerful tool for the investigation of neural networks in various animals. It also has potential as a visual prosthesis in case of photoreceptor degeneration [14]–[16].

Different modalities, such as pain, temperature and touch, are mixed when an animal senses the world through its skin. However, by the selective expression of ChR2 in a subset of nociceptive DRG neurons, the modality-specific circuitry has been optically investigated [17], [18]. We have recently generated several transgenic rat lines in which ChR2 transgene is driven by the Thy-1.2 promoter [16]. In one of these lines, W-TChR2V4, some neurons were endowed with photosensitivity by this introduction of ChR2; specifically, these neurons were the retinal ganglion cells, the principal neurons in the cerebral cortex and hippocampus, as well as other brain regions. (Figure S1). In this study, we identified that ChR2 is expressed in a certain population of large neurons in the DRG of a rat from this line. On the basis of their morphology and molecular markers, these neurons were classified as mechanoreceptive but not nociceptive. ChR2 was also found to be distributed in their peripheral sensory nerve endings. As the blue light evoked sensory nerve responses through the skin, it appeared to induce the sense of touch in the rats. It is thus suggested that the sensory modality of the somatosensory system was modified to induce reactivity to blue light in these transgenic rats.

## Results

### ChR2 expression in DRG

**Figure 1:**
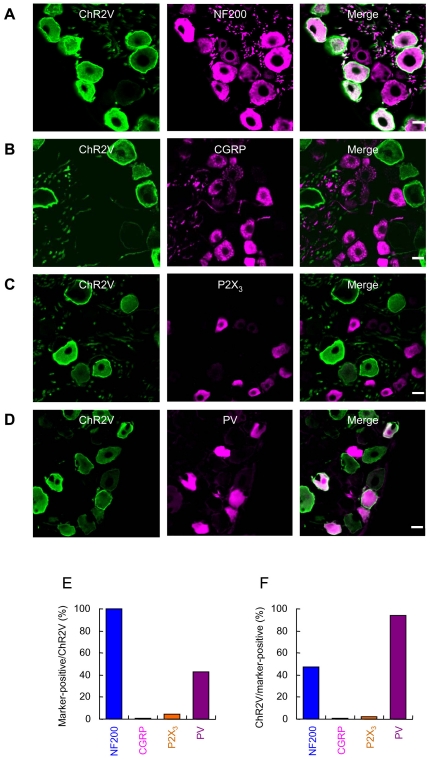
Distribution of channelrhodopsin 2-*Venus* conjugates (ChR2V) in the dorsal root ganglion (DRG) of W-TChR2V4 rats. A–D. Immunohistochemical identification of ChR2V-expressing neurons using cell-type specific markers, NF200 (A), CGRP (B), P2X_3_ (C) and PV (D). Scale bars indicate 20 µm. E. Probability of the co-expression of each marker, NF200, CGRP, P2X_3_ or PV, in the ChR2V+ neurons. F. Probability of the co-expression of ChR2V in the neurons positive for each marker, NF200, CGRP, P2X_3_ or PV.

The distribution of ChR2-*Venus* conjugates (ChR2V) was immunohistochemically identified using the W-TChR2V4 line. As shown in Figure 1A, the ChR2V-expressing (ChR2V+) DRG neurons always co-expressed NF200 (111/111 neurons, 100%, Figure 1E), one of the markers of myelinated neurons. On the other hand, almost negligible numbers of the ChR2V+ DRG neurons were positive for calcitonin gene-related peptide (CGRP) (3/279 neurons, 1.1%, Figure 1E) (Figure 1B) or P2X_3_ (7/161 neurons, 4.3%, Figure 1E) (Figure 1C). Previous studies have shown that some of the myelinated A fibers are also involved in proprioception [19], [20]. Parvalbumin (PV), a member of the family of low-molecular-weight calcium-binding proteins, has been shown to be preferentially expressed within large DRG neurons and is considered to be a highly specific molecular marker for primary proprioceptors [21], [22]. As shown in Figure 1D, some of the ChR2V+ DRG neurons co-expressed PV (108/253 neurons, 43%, Figure 1E). Although not all NF200-positive neurons were ChR2V+ (111/236, 47%), most of the PV-positive neurons were ChR2V+ (108/115 neurons, 94%) (Figure 1F).

**Figure 2:**
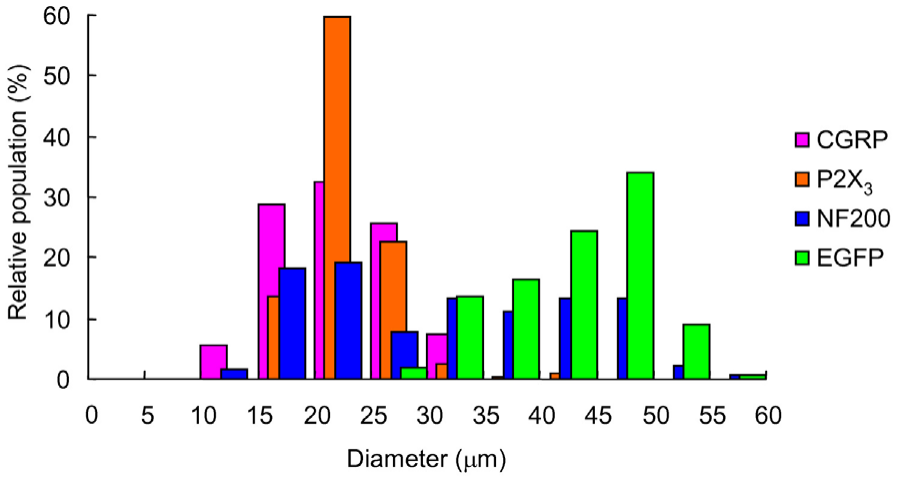
The average diameters of DRG neurons were compared among four groups positive for ChR2V (green), NF200 (blue), CGRP (magenta) and P2X_3_ (orange).

The size of each DRG neuron was evaluated by its average diameter, as summarized in Figure 2. The ChR2V+ DRG neurons (diameter, 43±0.42 µm, n = 212) were clearly discriminated in terms of size from the CGRP-positive DRG neurons (diameter, 23±0.34 µm, n = 230), with a statistically significant difference (P≪10^−10^, two-tailed *t*-test). Their size distribution was also different from that of the P2X_3_-positive DRG neurons (diameter, 23±0.24 µm, n = 229; P≪10^−10^, two-tailed *t*-test), although three P2X_3_-positive neurons had diameters between 35 and 45 µm. On the other hand, there was no significant difference in size between the CGRP- and the P2X_3_-positive groups. The NF200-positive neurons (n = 236) appeared to consist of at least two groups, one with diameters smaller than 30 µm and the other with diameters larger than 30 µm. The ChR2V+ DRG neurons were segregated from the former group but co-localized with the latter.

### ChR2 expression in dorsal spinal cord

**Figure 3:**
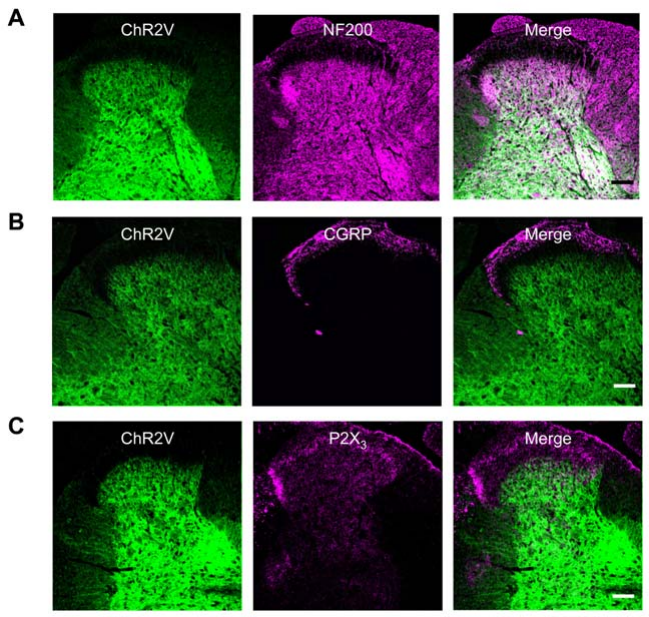
Distribution of ChR2V in the dorsal part of the spinal cord. A–C. Immunohistochemical localization of ChR2V with the cell-type specific markers, NF200 (A), CGRP (B) or P2X_3_ (C). Scale bars indicate 40 µm.

In the dorsal spinal cord, the gray matter has been anatomically classified into five discrete layers [23]. As shown in Figure 3A–C, the ChR2V was broadly distributed in the spinal cord gray matter (see also Figure S2). However, it was negligible in the outer dorsal layers where CGRP immunoreactivity was present (Figure 3B). Similarly, the ChR2V was not co-localized with P2X_3_ immunoreactivity in the inner dorsal layers (Figure 3C). On the other hand, the distribution of ChR2V overlapped with that of NF200 immunoreactivity (Figure 3A).

### ChR2 expression in the peripheral nerve endings

**Figure 4:**
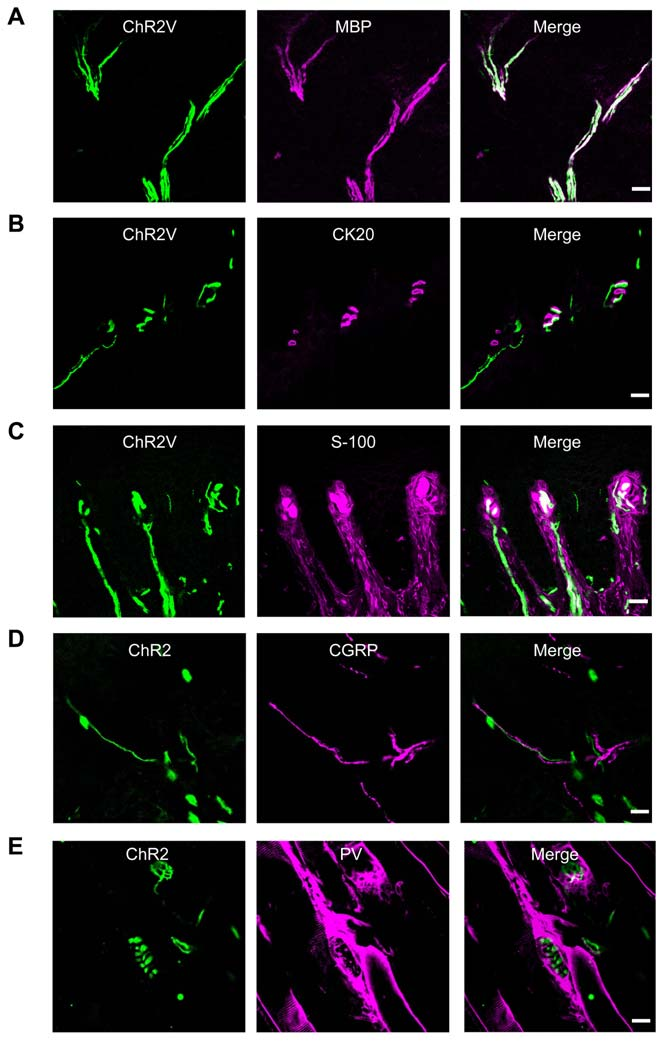
Distribution of ChR2V in the peripheral sensory nerve endings. A–D. Immunohistochemical identification of the ChR2V+ nerve endings in the skin in relation to MBP (A), CK20 (B), S-100 (C) or CGRP (D). E. Co-localization of the ChR2V+ nerve endings with PV in the muscle spindle. Scale bars indicate 20 µm.

As previously noted, ChR2 was expressed in large DRG neurons, which have been suggested to be involved in proprioception and touch-pressure sensing. The above results showed that only a subpopulation of ChR2V+ DRG neurons co-expressed PV, a marker of proprioceptive neurons. Therefore, it is probable that another subpopulation of these neurons have myelinated nerves involved in touch-pressure, which project their peripheral endings to the skin as mechanoreceptors. In the superficial layer of the skin, indeed, the ChR2V+ nerve bundles were also positive for myelin basic protein (MBP), which is a marker of myelinated axons (Figure 4A). Some of the peripheral endings of mechanoreceptive neurons have been shown to be associated with Merkel cells, which specifically express cytokeratin-20 (CK20), and form Merkel corpuscles in the skin [24], or with lamellar cells of Meissner's corpuscles, which express S-100 [25]. As shown in Figures 4B and 4C, ChR2V+ nerve endings were frequently associated with CK20-positive Merkel cells or with S-100-positive cells to form structures morphologically reminiscent of Meissner's corpuscles. On the other hand, they were not co-localized with the CGRP-positive free nerve endings assumed to be involved in nociception (Figure 4D). Peripherally, some of the proprioceptive DRG neurons projected to the intrafusal muscles as sensory spiral endings. As shown in Figure 4E, ChR2V+ nerve endings were frequently found in muscle. Some of them were motor nerve terminals as the spinal motor neurons also expressed ChR2V (Figure S2). Others were found in the muscle spindles with spiral appearances and co-expressed PV (Figure 4E).

### Sensitivity to blue light

**Figure 5:**
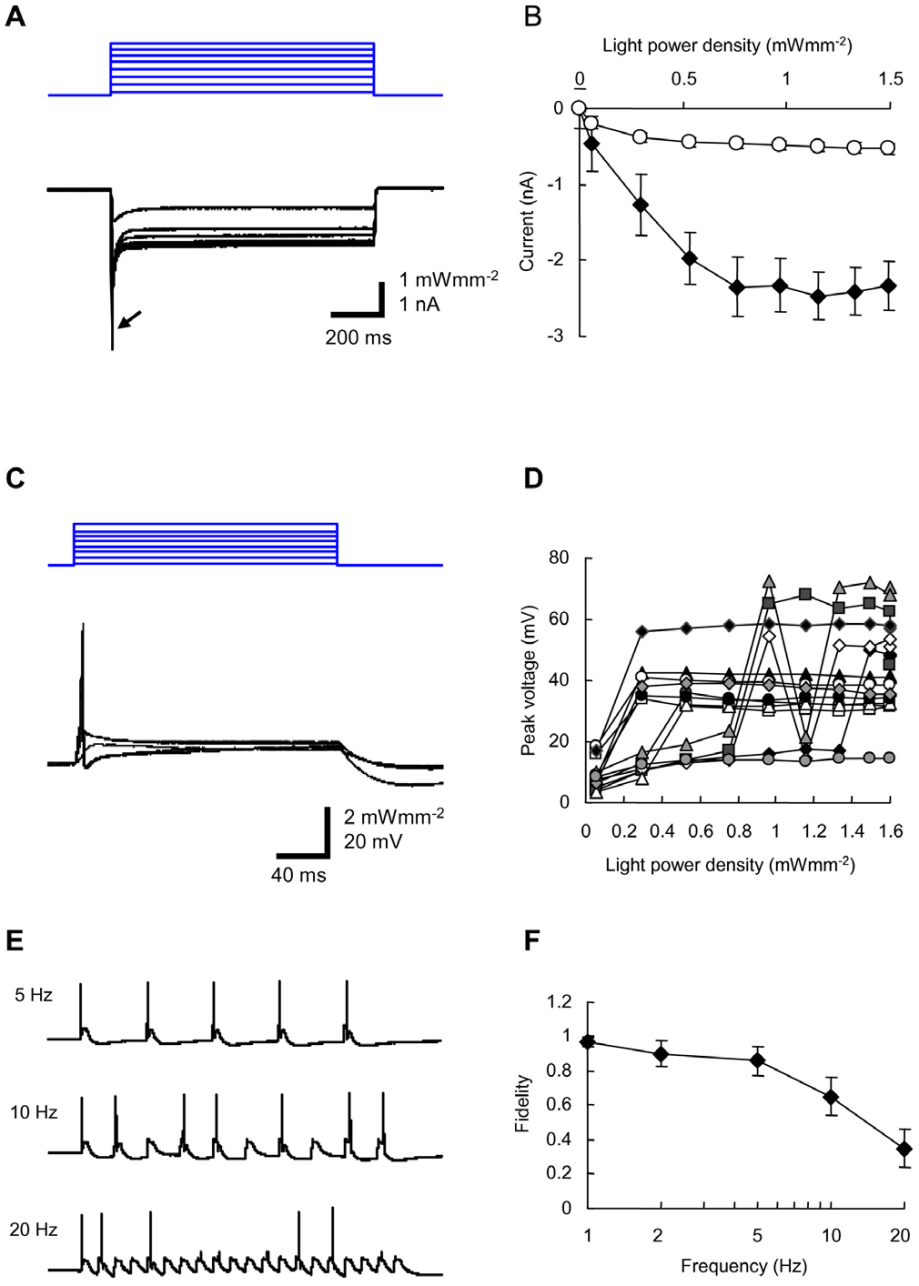
Optical responses of the ChR2V+ DRG neurons. A. Representative records of photocurrents (bottom) evoked by blue LED light of variable strength (top) under voltage clamp. An arrow indicates the unclamped current from escaped action potentials. B. The peak (closed diamonds) and the steady-state (open squares) photocurrent amplitudes (mean ± SEM, both n = 24) as functions of the light power density. C. Representative records of the neuronal membrane potential evoked by blue LED pulses (200 ms) of variable strength under current clamp. The resting membrane potential, −56 mV. D. The maximal voltage changes as a function of the light power density (n = 16). E. Representative records of membrane potential responses of a ChR2V-expressing DRG neuron to the repeated flashing of blue LED pulses (1.6 mWmm^−2^, 20 ms duration) at various frequencies, that is, 5 Hz (top), 10 Hz (middle) and 20 Hz (bottom). F. Fidelity of generation of action potentials as a function of frequency (mean ± SEM, n = 14).

We next evaluated the sensitivity of ChR2V+ DRG neurons to blue light. For this evaluation, the DRG neurons were cultured and the ChR2V expression was identified by the presence of *Venus* fluorescence. Under whole-cell voltage clamp, blue LED light pulse evoked a photocurrent in every ChR2V+ neuron (24/24 neurons) (Figure 5A). Both the peak and the steady-state photocurrents were dependent on the light power density (Figure 5B). The peak current ranged between −0.5 and −5.2 nA (n = 24) at the maximal irradiance, although unclamped currents from escaped action potentials were frequently observed. Under the current clamp, the blue LED light pulse (200 ms) evoked rapid membrane depolarization in an intensity-dependent manner and, eventually, only one action potential in 16 of 18 experiments (Figure 5C). In the remaining two cases, the blue LED light evoked subthreshold depolarization even at the maximum irradiance. The size of the action potential varied from cell to cell (range, 15–72 mV, n = 16) with threshold irradiances of 0.06–1.3 mWmm^−2^ (Figure 5D). However, the same blue LED light did not evoke any current or voltage response in the ChR2V-negative (ChR2V−) DRG neurons (n = 3, Figure S3).

When short LED pulses (duration, 20 ms) were repeatedly applied at the maximal irradiance (1.6 mWmm^−2^), they robustly evoked action potentials at low frequency (Figures 5E and 5F). For example, there was no failure of the action potential in 13/14 neurons at 1 Hz, 12/14 at 2 Hz and 10/14 at 5 Hz. However, the fidelity was reduced at increased frequencies: 6/14 at 10 Hz and 3/14 at 20 Hz.

### Behavioral responses to light

**Figure 6:**
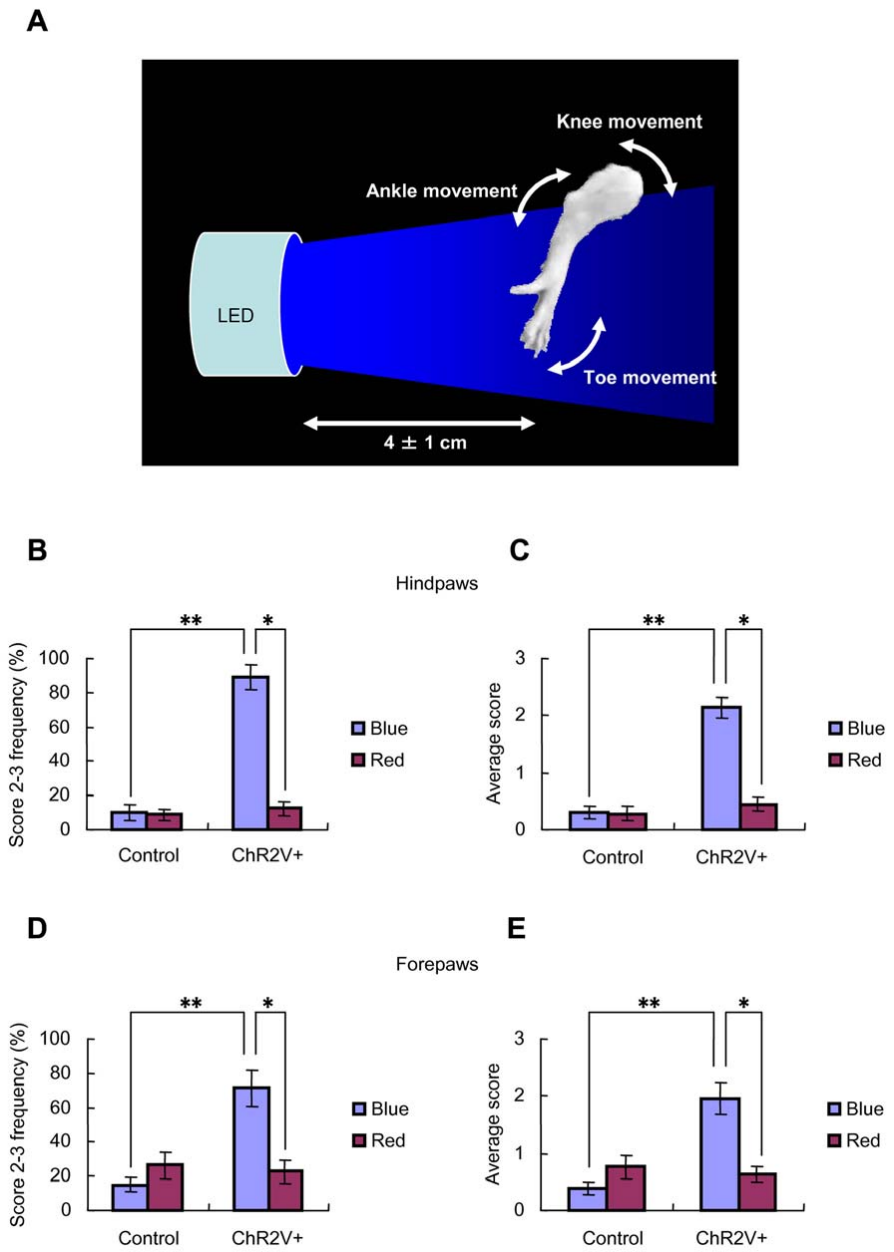
Optically evoked behavioral responses. A. Schematic diagram of the experimental setup. One of four paws was placed in the light path of a blue or red LED so that the plantar skin faced the light while the rest of the body was covered with a black cloth. The distance between the surface of the collimator lens of the LED and the plantar skin was set to about 4 cm, but the exact distance was in the range of 3–5 cm because of the spontaneous movements of the paw. All the experiments were carried out under yellow, dim background light. The optically evoked behavioral responses were scored according to the movement around joints: toe, ankle/wrist or knee/elbow (Table 1). B and C. Light-evoked responses of hindpaws were compared between the wild-type (n = 8) and the ChR2V+ rats (n = 8). The probability of large movements (score 2–3, B) or the average score over ten successive tests (C) was compared between blue and red LED light. D and E. Light-evoked responses of forepaws of the same group of rats: the probability of large movements (score 2–3, D) or the average score (E). Wicoxon signed-rank test was applied for the paired data (*, P<0.01) and Mann-Whitney *U*-test for unpaired data (**, P<0.0005).

On the basis of the above evidence, we could expect that the blue light on the plantar skin evokes tactile perception in this transgenic rat. To test this, hindpaws of rats were illuminated at the plantar skin by a series of blue or red LED flashes (duration, 50 ms; 10 pulses at 10 Hz; interval, 10 s) while the rest of the body was shaded with a black cloth (Figure 6A). Although the rats remained quiet, the ChR2V+ rats appeared to move the paw in response to the blue LED flash on its plantar skin (Video S1). The blue LED flashes clearly and robustly evoked reflexive movements of the paw or toe, whereas the red LED flashes did not (Video S2). The movements during flashing exposure were scored according to their magnitudes (Table 1). The proportion of cases when movement occurred during flashing with blue light was 100%, whereas it was 25±68% with red light, showing a significant difference (P<0.05, Wilcoxon signed-rank test, n = 8 animals). Relatively large movements (score, 2–3) were frequently evoked by the blue LED flashes (Figure 6B). There was a significant difference between blue and red flashes (n = 8, P<0.01, Wilcoxon signed-rank test) when we compared the average scores given for the magnitude of movement (Figure 6C). As a control, neither blue nor red flashes evoked clear movements in the case of ChR2V− rats (n = 8). Compared with the ChR2V+ rats, the probability of showing large movements (score 2–3) and the average movement scores of the control rats were negligible (Figures 6B and 6C, P<0.005, Mann-Whitney *U*-Test). Similarly, the ChR2V+ rats moved their forepaws significantly more frequently and vigorously during blue LED flash than during red LED flashes (Figures 6D and 6E; Videos S3 and S4). We also tested the light-evoked movement of tails, but only one ChR2V+ rat showed clear responses to the blue LED flashes (Video S5).

**Table 1:** The behavioral response score.

Behavior	Score
No response	0
Toe movements	1
Ankle or wrist movements	2
Knee or elbow movements	3

## Discussion

In this study, we found that ChR2 is expressed in a certain population of large neurons in the DRG of a transgenic rat line, W-TChR2V4, in which the ChR2V transgene was driven by the Thy-1.2 promoter [16]. The ChR2V+ neurons co-expressed NF200, a marker of medium–large neurons involved in mechanoreception, but not CGRP or P2X_3_, markers of small neurons involved in nociception. Since the peripheral sensory nerve endings also expressed ChR2, it was expected that photostimulation would evoke action potentials in them. Indeed, the ChR2V+ rats showed sensory-evoked behaviors in response to blue LED flash on their plantar skin. It is thus suggested that the rats had acquired an unusual sensory modality enabling them to sense blue light through the skin.

### ChR2V+ DRG neurons are not involved in nociception

The DRG comprises cell bodies of functionally distinct sensory nerves. The DRG neurons have been classified in terms of various characteristics, for example, fast-conducting vs. slow-conducting, myelinated vs. non-myelinated, nociceptive vs. non-nociceptive or small vs. large. In this study, we used NF200 as a marker of myelinated neurons and found that almost all ChR2V+ DRG neurons co-expressed NF200. On the other hand, some NF200-expressing DRG neurons were ChR2V−. It is thus suggested that the ChR2V-expressing DRG neurons are a subpopulation of the fast-conducting myelinated neurons.

The nociceptive neurons can be further classified into peptidergic and non-peptidergic neurons. The peptidergic neurons are immunoreactive to substance P (SP) or CGRP, whereas the non-peptidergic neurons are immunoreactive to isolectin B4 (IB4) or purinergic receptor P2X_3_ [26]–[28]. We found that almost all the ChR2V+ neurons were negative for both CGRP and P2X_3_. Therefore, the nociceptive DRG neurons appeared to be mostly ChR2V−.

In our study over 98% of the ChR2V+ DRG neurons had diameters larger than 30 µm. Although the neuron size is dependent on the age or the body size of the rat, these neurons were classified as medium–large [29]–[31]. On the other hand, the nociceptive neurons that expressed either CGRP or P2X_3_ were mostly small. The size distribution of ChR2V+ neurons appeared to be distinct from that of nociceptive neurons. However, a small number of medium-sized DRG neurons were also positive for P2X_3_. A subpopulation of NF200-expressing DRG neurons were discriminated from ChR2V+ DRG neurons by their small size (diameter<30 µm). It is possible that these DRG neurons were small myelinated Aδ-fiber neurons involved in pain [32]. These observations are consistent with the notion that the ChR2V+ neurons are not involved in nociception [33], [34], although further studies are necessary to confirm this.

In the spinal cord, neurons involved in nociception are located in the marginal layer (lamina I) and the substantia gelatinosa (lamina II) of the dorsal horn and receive inputs from the nociceptive DRG neurons [35], [36]. Indeed, various noxious stimuli induce acute c-Fos expression in these laminae [37]. On the other hand, the myelinated fibers involved in mechanoreception are projecting predominantly to laminae III–V [35]–[38]. Consistent with these previous studies, immunoreactivity to CGRP or P2X_3_ was predominantly present in laminae I and II and that to NF200 was negligible in these superficial layers. We found that the ChR2V expression co-localized with neither with CGRP nor P2X_3_. It is thus suggested that the ChR2V+ fibers are myelinated and involved in mechanoreception.

We also found in the plantar skin that the ChR2V+ nerve fibers were mostly associated with MBP immunoreactivity. It is thus suggested that these fibers were myelinated and distinct from the nociceptive C-fibers that were unmyelinated. They are also unlikely to be nociceptive Aδ fibers that had lost their myelin before entering the skin [39]. On the other hand, ChR2V-positive nerve endings were frequently associated with Merkel cells in the dermis or with S-100-positive cells to form structures like Meissner's corpuscles, suggesting their involvement in the sense of touch-pressure. However, we did not find the axon terminals in the deep mechanoreceptive structures such as Pacinian corpuscles; this was probably because our histological studies were limited to the superficial layer of the plantar skin. Some of the ChR2V+ fibers appeared to be involved in proprioception since they innervated the muscle spindle to form stretch receptors that were PV-positive.

Taken together, the above histological characteristics suggest that ChR2V was not expressed in most nociceptive DRG neurons. Rather, it appeared to be expressed in a subpopulation of mechanoreceptive neurons with myelinated fibers [40]. Some of the ChR2V+ neurons appeared to be involved in proprioception in muscles, tendons and joints since they were also positive for PV. However, others may have innervated the skin and been involved in the sense of touch-pressure.

### Light-evoked somatosensory responses

The above findings that the mechanoreceptive DRG neurons expressed ChR2V in both the soma and peripheral endings raised the possibility that the ChR2V+ rats can sense blue light on their skin as if it were a touch. There is further evidence to support this. First, the ChR2V+ DRG neurons were photosensitive and optically depolarized to evoke action potentials. The action potential was generated at the onset of the light pulse and not thereafter, a trait frequently found in mechanoreceptive DRG neurons [41]–[45]. Second, the ChR2V+ rats moved their paws in response to the blue LED flashes on the plantar skin, but not in response to the red LED flashes. This behavior was not observable in the control ChR2V− rats. Therefore, the light-evoked behaviors were dependent on both the expression of ChR2 and the wavelength (470±17 nm) that is optimal for the activation of ChR2 [9]. As ChR2V was exclusively expressed in the myelinated nerve endings that were non-nociceptive, the blue light was likely to have induced the sense of touch-pressure in the rats. This blue light-evoked response was not clear in other parts of the body such as the tail. It is possible that fur covering the skin would obstruct the penetration of light. Although ChR2V was expressed in motor neurons and their terminals in our rat model (Figure S2), the sensory nerve endings, which lie in the superficial layer of the skin, are expected to be differentially photostimulated because the blue light cannot penetrate deep into the muscle. It is thus suggested that the sensory modality of the somatosensory system was modified so as to be also reactive to blue light in the ChR2V+ rats.

### Conclusions

In this work, we have generated an optogenetic rat model that can be used for the research of the somatosensory system. Using ChR2V+ rats, we can discretely photostimulate the mechanoreceptive nerve endings without any effects on the nociceptive free nerve endings. Combined with electrophysiological as well as neuroimaging methods such as fMRI, our rat model should facilitate study of how complex tactile perception, such as for texture, size and shape, is generated.

## Materials and Methods

### Ethics Statement

All animal experiments were approved by the Tohoku University Committee for Animal Experiments (Approval No. 2011LsA-23) and were carried out in accordance with the Guidelines for Animal Experiments and Related Activities of Tohoku University as well as the guiding principles of the Physiological Society of Japan and the National institutes of health (NIH), USA.

### Animals

The experiments were carried out using offspring of one of the Thy-1 promotor-ChR2-*Venus* transgenic rat lines, W-TChRV4 with the background of Wistar rats [16] mated with wild-type Wistar rat. The littermates were screened by genomic PCR using the appropriate primers (Figure S4), and were determined to be either transgene-positive (ChR2V+) or -negative (ChR2V−). Alternatively, the tip of the tail was freshly examined under fluorescent microscopy to determine whether *Venus* fluorescence was present in the nerve bundle in the tissue (Figure S5). The number of animals in this study was kept to a minimum and, when necessary, all animals were anesthetized to minimize their suffering. Animals had access to food and water ad libitum and were kept under a 12-hour light-dark cycle.

### Immunohistochemistry

ChR2V+ rats (five weeks old) were used for the immunohistochemical experiments. They were anesthetized with a ketamine (50 mg/ml, Daiichi Sankyo Co. Ltd., Tokyo, Japan)-xylazine (xylazine hydrochloride, 10 mg/ml, Sigma-Aldrich, St. Louis, MO, USA) mixture (1 ml/kgBW) and transcardially perfused with phosphate-buffered saline (PBS; pH 7.4), followed by 100 ml of 4% paraformaldehyde (PFA) and 0.2% picric acid in PBS. The lumber region of spinal cords together with DRGs, the intercostals muscles and the pedal skin from one of the paws were removed and post-fixed in 4% PFA overnight at 4°C. After cryoprotection through a graded series of sucrose replacements (10%, 20% and 30% in PBS) at 4°C, each tissue was embedded in OCT Compound (4583, Sakura Finetek, Tokyo, Japan) and stored at −80°C.

The localization and cell type of ChR2V-expressing neurons in the tissue were immunohistochemically investigated using anti-EGFP antibody along with the antibody of one of the cell-type-specific markers. Briefly, each frozen section was cut at 16 µm thickness with a cryostat (CM 3050 S, Leica, Wetzlar, Germany), mounted on poly-l-lysine coated slides (Matsunami Glass Ind. Ltd., Kishiwada, Japan) and left to air-dry for 90 min at room temperature. After washing with PBS, slices were incubated for 1 hr in blocking PBS containing 2.5% goat serum, 0.25% carrageenan and 0.1% Triton X-100 at room temperature. Then, the specimens were reacted overnight at 4°C with the primary antibody: rabbit anti-EGFP (1∶2,000) [46]; mouse monoclonal anti-NF200 (1∶500, N0142, Sigma-Aldrich); rabbit anti-CGRP (1∶2,000, C8198, Sigma-Aldrich); guinea-pig anti-CGRP (1∶1000, Progen Biotechnik GmbH, Heidelberg, Germany); guinea-pig anti-P2X_3_ (1∶1,000, GP10108, Neuromics, Edina, MN, USA); mouse anti-PV (1∶2,000, P3088, Sigma-Aldrich); chicken anti-MBP (1∶500, PA1-10008, Thermo Fisher Scientific K.K., Yokohama, Japan), mouse anti-CK20 (1∶20, IT-Ks 20.8, Progen Biotechnik GmbH) and mouse anti-S100 (β-subunit) (1∶1000, S2532, Sigma-Aldrich). In some specimens, the *Venus* fluorescence signal could be directly examined without any amplification as previously described [47], [48]. After 10-min washing three times, the slices were reacted for 1 hr (room temperature) or overnight (4°C) with the combination of the following secondary antibodies (Molecular Probes products from Life Technologies Co., Carlsbad, CA, USA, except for Dylight-549): Alexa Fluor 488-conjugated donkey anti-rabbit IgG (1∶500), Alexa Fluor 546-conjugated donkey anti-mouse IgG (1∶500), Alexa Fluor 546-conjugated donkey anti-rabbit IgG (1∶500) and Alexa Fluor 633-conjugated goat anti-guinea pig IgG (1∶500), and Dylight 549-conjugated goat anti-chicken IgY (Thermo Fisher Scientific K.K., 1∶100). After washing three times in PBS, the specimens were mounted with Permafluor (Thermo Fisher Scientific K.K.). Images were digitally captured under conventional confocal laser-scanning microscopy (LSM510META, Carl Zeiss, Oberkochen, Germany) and were corrected for brightness and contrast using LSM Image Browser version 3.2 (Carl Zeiss), Photoshop version 6.0 (Adobe Systems Inc, San Jose, CA, USA) and ImageJ (http://rsbweb.nih.gov/ij/). The diameter of a DRG neuron was microscopically measured as the mean of the shortest and longest diameters.

### Culture

The DRG neurons of ChR2V+ rats (3–4 weeks) were cultured according to a method reported previously [49] with some modifications. After decapitation, the DRGs from all available spinal levels were taken out and put into an ice-cold dissecting solution. Each DRG was cleaned of the surrounding connective tissue, cut into small pieces and immersed in an enzymatic solution containing 1.0 mg/ml collagenase II (C6885, Sigma-Aldrich), 0.5 mg/ml trypsin (15090-046, a Gibco product from Life Technologies Co.) and 0.1 mg/ml DNase I (Sigma-Aldrich) for 30–45 min at 37°C. The neurons were washed twice with trituration solution containing 2 mg/ml BSA (A7906, Sigma-Aldrich), resuspended in culture medium containing DMEM (D5030, Sigma-Aldrich) supplemented with 3.7 mg/ml NaHCO_3_, 1 mg/ml d-glucose, 2 mM l-glutamine (G7513, Sigma-Aldrich), 1% penicillin/streptomycin (P0781, Sigma-Aldrich) and 10% FBS (04-001-1, Biological Industries, Beit-Haemek, Israel), and then plated and cultured at 37°C in a humidified incubator with a 95% air and 5% CO_2_ atmosphere. The culture medium was changed every two days. The whole-cell recording experiments were carried out within 4–5 days of plating.

### Electrophysiology

ChR2V+ DRG neurons were identified under conventional epi-fluorescence microscopy (BH2-RFC, Olympus Optical Co., Tokyo, Japan) equipped with a 40× water-immersion objective (LUMplanP1/IR40×, Olympus) and a conventional filter cube (excitation, 495 nm; dichroic mirror, 505 nm; barrier filter, 515 nm). Electrophysiological recording was performed at 34±2°C (UTC-1000, Ampere Inc., Tokyo, Japan) under whole-cell patch clamp from the soma using an amplifier (EPC 8, HEKA Elektronik Dr. Schulze GmbH, Germany) and computer software (pCLAMP 9, Molecular Devices, LLC, Sunnyvale, CA). The bath solution was composed of (in mM) 138 NaCl, 3 KCl, 2 CaCl_2_, 1 MgCl_2_, 4 NaOH, 10 HEPES, 11 glucose, and was adjusted at pH 7.4 by 1 N HCl. The patch pipette solution was composed of (in mM) 125 K-gluconate, 10 KCl, 0.2 EGTA, 10HEPES, 1 MgCl_2_, 3 MgATP, 0.3Na_2_GTP, 10 Na_2_-phosphocreatine and 0.1 leupeptin, and was adjusted at pH 7.2 by 1 N KOH. For the optical actuation of a DRG neuron we used a blue LED (470±25 nm wavelength, LXHL-NB98, Philips Lumileds Lighting Co., San Jose, CA, USA) regulated by a pulse generator (SEN-7203, Nihon Kohden, Tokyo, Japan) and computer software (pCLAMP 9, Molecular Devices, LLC). The maximal light power density of the LED light was 1.6 mWmm^−2^ at the focus.

### Behavioral test

Light-dependent behavior was investigated using eight ChR2V-expressing (ChR2V+) (9–21 weeks) and eight non-expressing (ChR2V−) rats, including three littermates (8–14 weeks) and five wild-type Wistar rats (15 weeks). The whole body of a rat, except for one of the four paws and the tail, was shaded from the light with a black cloth. Either a blue (470±17 nm, LXML-PB01-0023, Philips Lumileds Lighting Co.) or a red LED (627±15 nm, LXML-PD01-0030, Philips Lumileds Lighting Co.) was driven by a pulse generator (SEN-7203, Nihon Kohden, Tokyo, Japan) and a DC voltage/current generator/calibrator (R6243, Advantest, Tokyo, Japan). A series of flashes (duration, 50 ms; 10 pulses at 10 Hz; interval, 10 s) was subjected to the skin at a distance of 3–5 cm while the behavior of the rat was captured using a video camera (PC1249, Canon, Tokyo, Japan). All experiments were carried out under yellow, dim background light. The light power density was directly measured using a thermopile (MIR-100Q, Mitsubishi Oil Chemicals, Tokyo, Japan), and was 3–8 mWmm^−2^ at the skin for both the blue and the red LED lights. Under double-blind conditions, the response to light was scored as described in Table 1.

## Supporting Information

Video S1 **Typical ChR2V+ transgenic rat showed sensory-evoked behavior of a hindpaw in response to a series of blue LED flashes on the plantar skin as if it had been touched.**
(MPG)

Video S2 **The same hindpaw showed no specific response to a train of red LED flashes.**
(MPG)

Video S3 **The same rat also showed sensory-evoked behavior of a forepaw in response to a train of blue LED flashes.**
(MPG)

Video S4 **The same forepaw showed no specific response to a train of red LED flashes.**
(MPG)

Video S5 **The same rat also showed clear sensory-evoked movement of its tail in response to a train of blue LED flashes as if it had been touched.**
(MPG)

Figure S1 **Expression of ChR2V in the brain of W-TChR2V4 rat.**
(PDF)

Figure S2 **Expression of ChR2V in the spinal cord and the motor nerve terminals.**
(PDF)

Figure S3 **Non-fluorescent DRG neurons were unresponsive to the blue light.**
(PDF)

Figure S4 **The primers that were used to differentiate ChR2V+ from ChR2V− rats.**
(PDF)

Figure S5 **Expression of ChR2V in the tail nerve bundles.**
(PDF)

## References

[1] Nathans J (1999). The evolution and physiology of human color vision: insights from molecular genetic studies of visual pigments.. Neuron.

[2] Palczewski K (2006). G protein-coupled receptor rhodopsin.. Annu Rev Biochem.

[3] Kaupp UB, Seifert R (2002). Cyclic nucleotide-gated ion channels.. Physiol Rev.

[4] Bradley J, Reisert J, Frings S (2005). Regulation of cyclic nucleotide-gated channels.. Curr Opin Neurobiol.

[5] Sineshchekov OA, Jung KH, Spudich JL (2002). Two rhodopsins mediate phototaxis to low and high intensity light in *Chlamydomonas reinhardtii*.. Proc Natl Acad Sci U S A.

[6] Suzuki T, Yamasaki K, Fujita S, Oda K, Iseki M (2003). Archaeal-type rhodopsins in *Chlamydomonas*: model structure and intracellular localization.. Biochem Biophys Res Commun.

[7] Kateriya S, Nagel G, Bamberg E, Hegemann P (2004). “Vision” in single-celled Algae.. News Physiol, Sci.

[8] Nagel G, Ollig D, Fuhrmann M, Kateriya S, Musti AM (2002). Channelrhodopsin-1: a light-gated proton channel in green algae.. Science.

[9] Nagel G, Szellas T, Huhn W, Kateriya S, Adeishvili N (2003). Channelrhodopsin-2, a directly light-gated cation-selective membrane channel.. Proc Natl Acad Sci U S A.

[10] Hegemann P (2008). Algal sensory photoreceptors.. Annu Rev Plant Biol.

[11] Boyden ES, Zhang F, Bamberg E, Nagel G, Deisseroth K (2005). Millisecond-timescale, genetically targeted optical control of neural activity.. Nat Neurosci.

[12] Li X, Gutierrez DV, Hanson MG, Han J, Mark MD (2005). Fast noninvasive activation and inhibition of neural and network activity by vertebrate rhodopsin and green algae channelrhodopsin.. Proc Natl Acad Sci U S A.

[13] Ishizuka T, Kakuda M, Araki R, Yawo H (2006). Kinetic evaluation of photosensitivity in genetically engineered neurons expressing green algae light-gated channels.. Neurosci Res.

[14] Bi A, Cui J, Ma YP, Olshevskaya E, Pu M (2006). Ectopic expression of a microbial-type rhodopsin restores visual responses in mice with photoreceptor degeneration.. Neuron.

[15] Tomita H, Sugano E, Yawo H, Ishizuka T, Isago H (2007). Restoration of visual response in aged dystrophic RCS rats using AAV-mediated channelopsin-2 gene transfer.. Invest Ophthalmol Vis Sci.

[16] Tomita H, Sugano E, Fukazawa Y, Isago H, Sugiyama Y (2009). Visual properties of transgenic rats harboring the channelrhodopsin-2 gene regulated by the Thy-1.2 promoter.. PLoS One.

[17] Campagnola L, Wang H, Zylka MJ (2008). Fiber-coupled light-emitting diode for localized photostimulation of neurons expressing channelrhodopsin-2.. J Neurosci Meth.

[18] Wang H, Zylka MJ (2009). *Mrgprd*-expressing polymodal nociceptive neurons innervate most known classes of substantia gelatinosa neurons.. J Neurosci.

[19] Lawson SN, Waddell PJ (1991). Soma neurofilament immunoreactivity is related to cell size and fibre conduction velocity in rat primary sensory neurons.. J Physiol.

[20] Julius D, Basbaum AI (2001). Molecular mechanisms of nociception.. Nature.

[21] Celio MR (1990). Calbindin D-28k and parvalbumin in the rat nervous system.. Neuroscience.

[22] Ichikawa H, Deguchi T, Nakago T, Jacobowitz DM, Sugimoto T (1994). Parvalbumin, calretinin and carbonic anhydrase in the trigeminal and spinal primary neurons of the rat.. Brain Res.

[23] Basbaum AI, Jessell TM, Kandel ER, Schwartz JH, Jessell TM (2000). The perception of pain.. Principles of Neural Science Fourth Edition.

[24] Moll I, Roessler M, Brandner JM, Eispert AC, Houdek P (2005). Human merkel cells-aspects of cell biology, distribution and functions.. Eur J Cell Biol.

[25] Kinnman E, Aldskogius H, Johansson O, Wiesenfeld-Hallin Z (1992). Collateral reinnervation and expansive regenerative reinnervation by sensory axons into “foreign” denervated skin: an immunohistochemical study in the rat.. Exp Brain Res.

[26] Averill S, Mcmahon SB, Clary DO, Reichardt LF, Priestley JV (1995). Immunocytochemical localization of trkA receptors in chemically identified subgroups of adult rat sensory neurons.. Eur J Neurosci.

[27] Snider WD, McMahon SB (1998). Tackling pain at the source: new ideas about nociceptors.. Neuron.

[28] Zhang X, Bao L (2006). The development and modulation of nociceptive circuitry.. Curr Opin Neurobiol.

[29] Ha SO, Yoo HJ, Park SY, Hong HS, Kim DS (2000). Capsaicin effects on brain-derived neurotrophic factor in rat dorsal root ganglia and spinal cord.. Brain Res Mol Brain Res.

[30] Fang X, Djouhri L, McMullan S, Berry C, Waxman SG (2006). Intense isolectin-B4 binding in rat dorsal root ganglion neurons distinguishes C-fiber nociceptors with broad action potentials and high Nav1.9 expression.. J Neurosci.

[31] Lu SG, Gold MS (2008). Inflammation-induced increase in evoked calcium transients in subpopulations of rat DRG neurons.. Neuroscience.

[32] Dawson LF, Phillips JK, Finch PM, Inglis JJ, Drummond PD (2011). Expression of α_1_-adrenoceptors on peripheral nociceptive neurons.. Neuroscience.

[33] Vulchanova L, Riedl MS, Shuster SJ, Stone LS, Hargreaves KM (1998). P2X_3_ is expressed by DRG neurons that terminate in inner lamina II.. Eur J Neurosci.

[34] Novakovic SD, Kassotakis LC, Oglesby IB, Smith JA, Eglen RM (1999). Immunocytochemical localization of P2X3 purinoceptors in sensory neurons in naïve rats and following neuropathic injury.. Pain.

[35] Caspary T, Anderson KV (2003). Patterning cell types in the dorsal spinal cord: what the mouse mutants say.. Nat Rev Neurosci.

[36] Morris R, Cheunsuang O, Stewart A, Maxwell D (2004). Spinal dorsal horn neurone targets for nociceptive primary afferents: do single neurone morphological characteristics suggest how nociceptive information is processed at the spinal level.. Brain Res Brain Res Rev.

[37] Coggeshall RE (2005). Fos, nociception and the dorsal horn.. Prog Neurobiol.

[38] Todd AJ (2002). Anatomy of primary afferents and projection neurons in the rat spinal dorsal horn with particular emphasis on substance P and the neurokinin 1 receptor.. Exp Physiol.

[39] Provitera V, Nolano M, Pagano A, Caporaso G, Stancanelli A (2007). Myelinated nerve endings in human skin.. Muscle Nerve.

[40] Perry MJ, Lawson SN, Robertson J (1991). Neurofilament immunoractivity in populations of rat primary afferent neurons: a quantitative study of phosphorylated and non-phosphorylated subunits.. J Neurocytol.

[41] Melean MJ, Bennett PB, Thomas RM (1988). Subtypes of dorsal root ganglion neurons based on different inward currents as measured by whole-cell voltage clamp.. Mol Cell Biochem.

[42] Villière V, McLachlan EM (1996). Electrophysiological properties of neurons in intact rat dorsal root ganglia classified by conduction velocity and action potential duration.. J Neurophysiol.

[43] Price MP, Lewin GR, McIlwrath SL, Cheng C, Xie J (2000). The mammalian sodium channel BNC1 is required for normal touch sensation.. Nature.

[44] Tan ZY, Donnelly DF, LaMotte RH (2006). Effects of a chronic compression of the dorsal root ganglion on voltage-gated Na^+^ and K^+^ currents in cutaneous afferent neurons.. J Neurophysiol.

[45] Hu J, Chiang LY, Koch M, Lewin GR (2010). Evidence for a protein tether involved in somatic touch.. EMBO J.

[46] Tamamaki N, Nakamura K, Furuta T, Asamoto K, Kaneko T (2000). Neurons in Golgi-stain-like images revealed by GFP-adenovirus infection in vivo.. Neurosci Res.

[47] Gong S, Zheng C, Doughty ML, Losos K, Didkovsky N (2003). A gene expression atlas of the central nervous system based on bacterial artificial chromosomes.. Nature.

[48] Torsney C, Anderson RL, Ryce-Paul KA, MacDermott AB (2006). Characterization of sensory neuron subpopulations selectively expressing green fluorescent protein in phosphodiesterase 1C BAC transgenic mice.. Mol Pain.

[49] Hu HZ, Li ZW (1997). Modulation by adenosine of GABA-activated current in rat dorsal root ganglion neurons.. J Physiol.

