# Supplementary material for: Light-evoked Somatosensory Perception of Transgenic Rats That Express Channelrhodopsin-2 in Dorsal Root Ganglion Cells
Source: PLoS One. 2012 Mar 6;7(3):e32699. doi: 10.1371/journal.pone.0032699 (PMC3295764; doi:10.1371/journal.pone.0032699)
Supplement: Figure S1 — Expression of ChR2V in the brain of W-TChR2V4 rat. (PDF) [file pone.0032699.s006.pdf]

Figure S1 Expression of ChR2V in the brain of W-TChR2V4 rat.

A

B

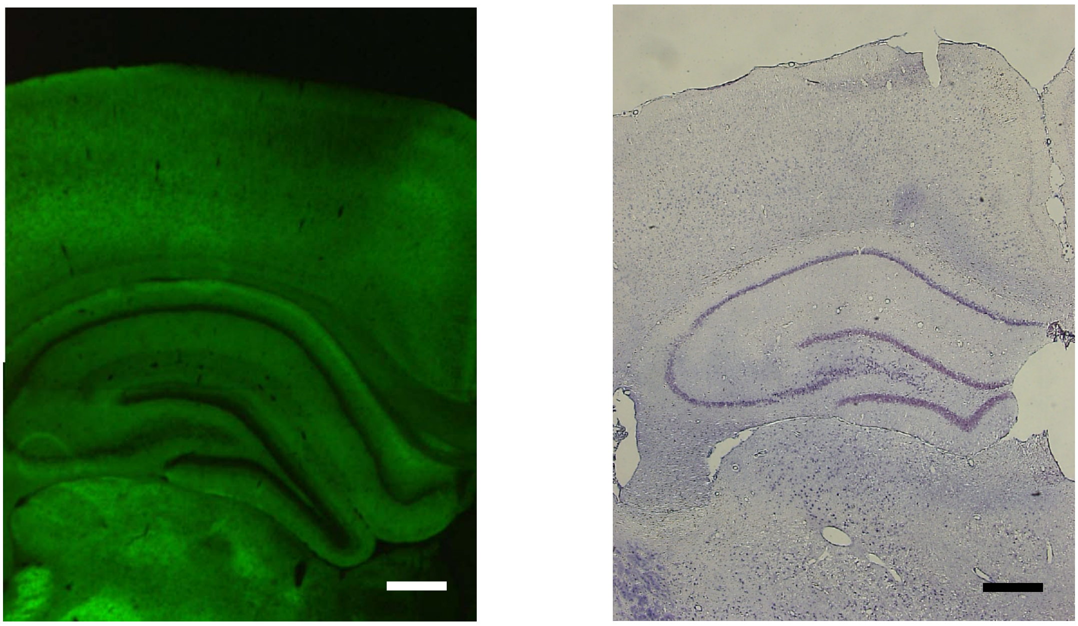

A. The distribution of Venus fluorescence in a coronal section of the brain of a W-TChR2V4 rat. **B.** *In situ* hybridization study of ChR2V mRNA. Note that the principal neurons of hippocampus are expressed with ChR2V mRNA in the nuclei, but appeared less fluorescent than the surrounding tissue. This is because the ChR2V molecules were exclusively distributed in the membrane of soma, dendrites and axons. Each scale bar indicates 500 μm.
