# Supplementary material for: Light-evoked Somatosensory Perception of Transgenic Rats That Express Channelrhodopsin-2 in Dorsal Root Ganglion Cells
Source: PLoS One. 2012 Mar 6;7(3):e32699. doi: 10.1371/journal.pone.0032699 (PMC3295764; doi:10.1371/journal.pone.0032699)
Supplement: Figure S2 — Expression of ChR2V in the spinal cord and the motor nerve terminals. (PDF) [file pone.0032699.s007.pdf]

Figure S2 Expression of ChR2V in the spinal cord and the motor nerve terminals.

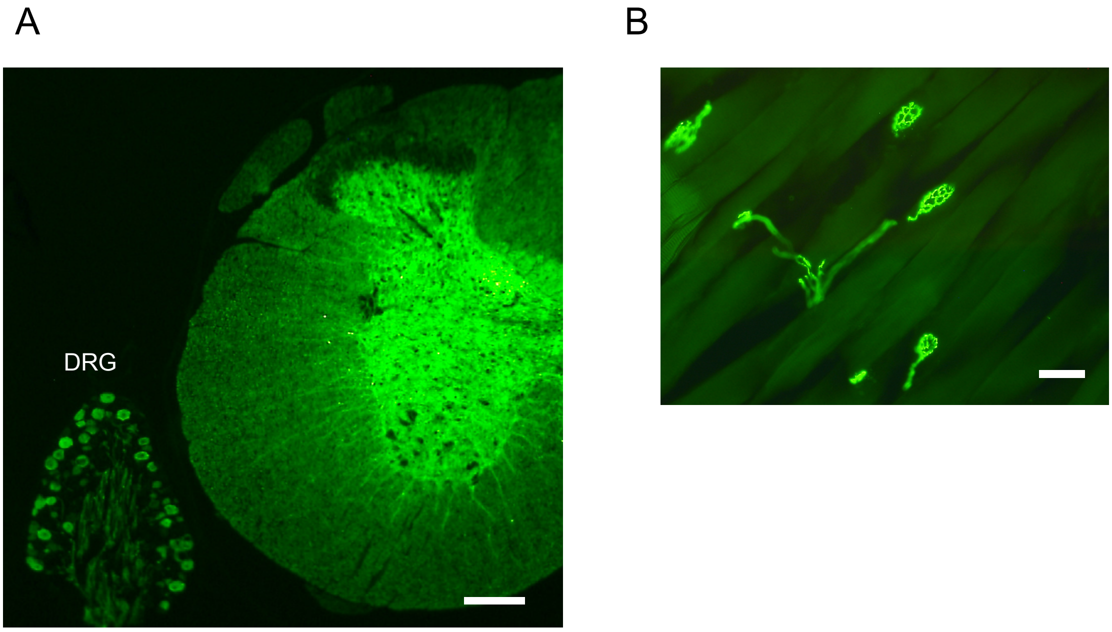

A. Transverse slices of spinal cord were immunohistochemically studied using anti-EGFP antibody with the Alexa Fluor 488-conjugated secondary anti-rabbit IgG. Note that the spinal motor neurons are expressed with ChR2V in the membrane of soma and axons. Scale, 200 μm. **B.** Intercostal musle. The motor nerve terminals are expressing ChR2V. Scale, 50 μm.
