# Supplementary material for: Light-evoked Somatosensory Perception of Transgenic Rats That Express Channelrhodopsin-2 in Dorsal Root Ganglion Cells
Source: PLoS One. 2012 Mar 6;7(3):e32699. doi: 10.1371/journal.pone.0032699 (PMC3295764; doi:10.1371/journal.pone.0032699)
Supplement: Figure S3 — Non-fluorescent DRG neurons were unresponsive to the blue light. (PDF) [file pone.0032699.s008.pdf]

Figure S3 Non-fluorescent DRG neurons were unresponsive to the blue light.

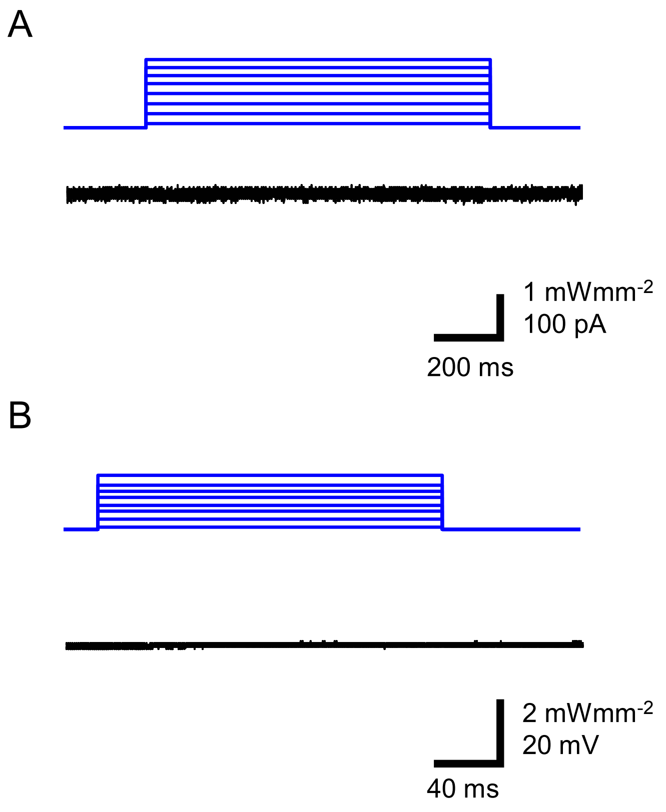

A. Sample records of photocurrents (bottom) during blue LED light pulses (1 s) of variable strength (top) under voltage clamp. **B.** The membrane potential during the blue LED pulses (200 ms) of variable strength under current clamp. From the same neuron in A. The resting membrane potential, -59 ~ -63 mV.
