# Supplementary material for: Light-evoked Somatosensory Perception of Transgenic Rats That Express Channelrhodopsin-2 in Dorsal Root Ganglion Cells
Source: PLoS One. 2012 Mar 6;7(3):e32699. doi: 10.1371/journal.pone.0032699 (PMC3295764; doi:10.1371/journal.pone.0032699)
Supplement: Figure S4 — The primers that were used to differentiate ChR2V+ from ChR2V− rats. (PDF) [file pone.0032699.s009.pdf]

Figure S4 The primers that were used to differentiate ChR2V+ from ChR2V- rats..

Venus-F: 5’-ATGGTGAGCAAGGGCGAGGAGCTGT-3’ (25-mer)

Venus-R: 5’-TTACTTGTACAGCTCGTCCATGCCGA-3’ (26-mer)
