# Supplementary material for: Light-evoked Somatosensory Perception of Transgenic Rats That Express Channelrhodopsin-2 in Dorsal Root Ganglion Cells
Source: PLoS One. 2012 Mar 6;7(3):e32699. doi: 10.1371/journal.pone.0032699 (PMC3295764; doi:10.1371/journal.pone.0032699)
Supplement: Figure S5 — Expression of ChR2V in the tail nerve bundles. (PDF) [file pone.0032699.s010.pdf]

Figure S5 Expression of ChR2V in the tail nerve bundles.

A

B

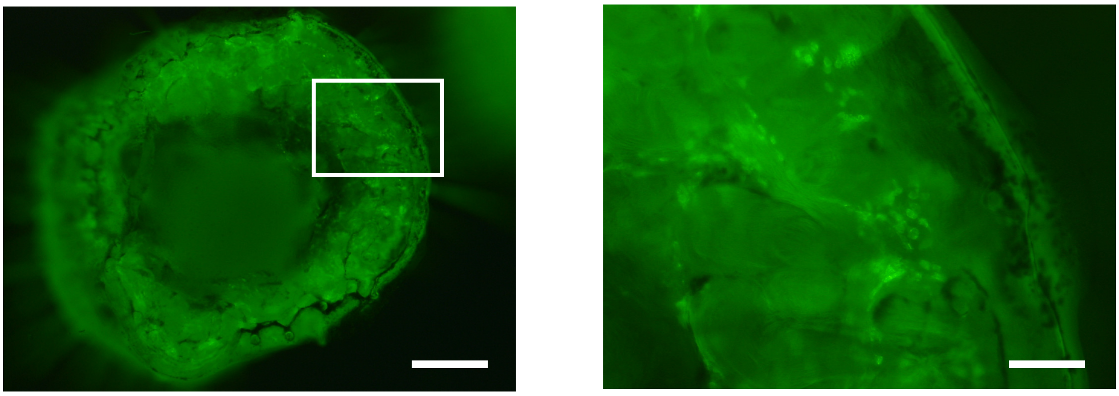

A. The Venus fluorescence in a fresh cross section of the tail of a 3-weeks old W-TChR2V4 rat. **B.** Enlarged view of the region indicated by a square in A. Note that the nerve bundles are fluorescent. Scale bars, 400 μm (A) and 100 μm (B).
